# Transcranial magnetic stimulation in anxiety and trauma‐related disorders: A systematic review and meta‐analysis

**DOI:** 10.1002/brb3.1284

**Published:** 2019-05-07

**Authors:** Patricia Cirillo, Alexandra K. Gold, Antonio E. Nardi, Ana C. Ornelas, Andrew A. Nierenberg, Joan Camprodon, Gustavo Kinrys

**Affiliations:** ^1^ Department of Psychiatry Massachusetts General Hospital Boston Massachusetts; ^2^ Division of Neuropsychiatry, Department of Psychiatry Massachusetts General Hospital Charlestown Massachusetts; ^3^ Universidade Federal do Rio de Janeiro, Rio de Janeiro Brazil; ^4^ Department of Psychological and Brain Sciences Boston University Boston Massachusetts; ^5^ Dauten Family Center for Bipolar Treatment Innovation Massachusetts General Hospital Boston Massachusetts; ^6^ Harvard Medical School Boston Massachusetts

**Keywords:** anxiety disorders, posttraumatic stress disorder, meta‐analysis, systematic review, theta burst, transcranial magnetic stimulation

## Abstract

**Background:**

Transcranial magnetic stimulation (TMS) has been evaluated as an effective treatment option for patients with major depressive disorder. However, there are limited studies that have evaluated the efficacy of TMS for other neuropsychiatric disorders such as anxiety and trauma‐related disorders. We reviewed the literature that has evaluated TMS as a treatment for anxiety and trauma‐related disorders.

**Methods:**

We searched for articles published up to December 2017 in Embase, Medline, and ISI Web of Science databases, following the Preferred Items for Reporting of Systematic Reviews and Meta‐Analyses (PRISMA) statement. Articles (*n* = 520) evaluating TMS in anxiety and trauma‐related disorders were screened and a small subset of these that met the eligibility criteria (*n* = 17) were included in the systematic review, of which nine evaluated TMS in posttraumatic stress disorder (PTSD), four in generalized anxiety disorder (GAD), two in specific phobia (SP), and two in panic disorder (PD). The meta‐analysis was performed with PTSD and GAD since PD and SP had an insufficient number of studies and sample sizes.

**Results:**

Among anxiety and trauma‐related disorders, TMS has been most widely studied as a treatment for PTSD. TMS demonstrated large overall treatment effect for both PTSD (ES = −0.88, 95% CI: −1.42, −0.34) and GAD (ES = −2.06, 95% CI: −2.64, −1.48), including applying high frequency over the right dorsolateral prefrontal cortex. Since few studies have evaluated TMS for SP and PD, few conclusions can be drawn.

**Conclusions:**

Our meta‐analysis suggests that TMS may be an effective treatment for GAD and PTSD.

**Highlights**

- We reviewed TMS as a treatment for anxiety disorders and PTSD.
- TMS presented large effect sizes as a treatment for PTSD and GAD.
- Follow‐up studies in GAD showed improvement of patients after TMS.
- Future studies should evaluate maintenance treatment.

## 1 INTRODUCTION

Transcranial magnetic stimulation (TMS) is a safe, effective, noninvasive, and nonconvulsive neuromodulation therapy cleared by the U.S. Food and Drug Administration (FDA) for the treatment of the major depressive disorder (MDD) since 2008 (O'Reardon et al., 2007) and for obsessive–compulsive disorder (OCD) since 2018. Other neurological and psychiatric conditions are being investigated as possible indications for TMS, including bipolar disorder, posttraumatic stress disorder (PTSD), chronic pain, and Alzheimer's disease, among others (Cotelli, Manenti, Cappa, Zanetti, & Miniussi, 2018; Lefaucheur et al., 2014; Nahas, Kozel, Li, Anderson, & George, 2003; Watts, Landon, Groft, & Young‐Xu, 2012).

TMS is a biomedical application of Faraday's principle of electromagnetic induction, and it works by generating strong and rapidly changing electric currents in a circular coil that is placed on the surface of the skull. This primary current generates a magnetic field that travels unimpeded through the hair, soft tissue, skull, and cerebrospinal fluid (i.e., these structures are minimally affected by the magnetic field) until it reached the neurons of the cortex. At this level, the magnetic field converts back into a (secondary) electrical current able to depolarize neurons and force an action potential, which will then travel from synapse to synapse across an entire functional circuit of interest (Camprodon & Pascual‐Leone, 2016). In a parameter‐dependent manner, TMS can induce long‐lasting plastic changes and can cause either a long‐term potentiation‐like effect or a long‐term depression‐like effect on cortical neurons, and this can modulate the physiological dynamics across brain regions and networks (Huerta & Volpe, 2009). In this context, TMS has the potential to therapeutically modulate aberrant circuit properties across neuropsychiatric conditions with maladaptive circuit dynamics. Recent technical development has introduced variants of the traditional repetitive TMS (rTMS) protocols such as deep TMS (dTMS) or theta burst stimulation (TBS), both with current FDA‐clearance for the treatment of OCD and MDD, respectively.

Anxiety and trauma‐related disorders include conditions related to maladaptive fear processing and related behavioral changes (Marin, Camprodon, Dougherty, & Milad, 2014). Anxiety is a broad clinical concept and occurs with different features in each disorder and individual, like the anticipation of future, sudden periods of intense fear with somatic sensations, or worry of being judged. The most prevalent anxiety disorders in adults are generalized anxiety disorder (GAD), panic disorder (PD) and agoraphobia, specific phobia (SP), and social anxiety disorder (SAD) (Bandelow & Michaelis, 2015). Before the Diagnostic and Statistical Manual of Mental Disorders, Fifth Edition (DSM‐5), PTSD was also considered an anxiety disorder (Association, 2000).

The lifetime comorbidity rates of PTSD with other psychiatric disorders range from 62% to 92% (Perkonigg, Kessler, Storz, & Wittchen, 2000). Furthermore, there is evidence that PD, GAD, and PTSD may have a common genetic predisposition (Chantarujikapong et al., 2001). There is a significant percentage of patients who suffer from these disorders and show no improvement after several trials with pharmacotherapy and cognitive behavior therapy (Ballenger et al., 2004). This highlights the need to continue therapeutic development research for anxiety disorders, and to consider the role of device‐based interventions such as TMS. The objective of this systematic review is to review and evaluate the existing literature on TMS for treating anxiety disorders and PTSD.

## 2 MATERIALS AND METHODS

### 2.1 Literature review

We screened Embase, PubMed, and ISI Web of Science (up to December 2017) following the recommendations of the Preferred Items for Reporting of Systematic Reviews and Meta‐Analyses (PRISMA) statement (Moher, Liberati, Tetzlaff, & Altman, 2009). The search terms used were (“TMS” OR “Repetitive TMS” OR “Transcranial Magnetic Stimulation” OR “theta‐burst”) AND (“Anxiety Disorders” OR “Social Anxiety” OR “Generalized Anxiety Disorder” OR “Panic disorder” OR “stress disorder, post‐traumatic” OR “Social, Phobia” OR “phobic disorder” OR "Phobia, Specific") NOT ("Obsessive‐Compulsive Disorder" OR "Anxiety, Separation" OR "Neurocirculatory Asthenia" OR "Neurotic Disorders"). We also examined the reference lists from selected articles in search of papers that could be missing. Only original articles published in English were included. Studies with animals and duplicated references were excluded.

### 2.2 Eligibility criteria and study selection

The eligibility criteria for the inclusion of studies in the present review were:

1. Treatment of SP, SAD, GAD, PD, or PTSD diagnosed according to DSM‐IV to DSM‐5 or ICD‐10 classifications.
2. Intervention with any form of TMS with at least five sessions (except for SP), because this is the minimum number of sessions to induce plasticity and improve symptoms for long term, while in SP a short‐term effect may be useful since the symptoms are more punctual (Racine, Chapman, Trepel, Teskey, & Milgram, 1995).
3. Report of response and remission rates, or score reduction on a validated scale of the investigated disorder.
4. Articles are written in English.

Controlled studies or open‐label studies with or without randomization and retrospective studies were accepted. Two researchers evaluated titles and abstracts to select potentially eligible articles, full papers were assessed to confirm eligibility whenever necessary, and divergences were solved by consensus.

### 2.3 Quality assessment and data extraction

The assessment of the quality of the studies and risk of bias followed the Cochrane guidelines (Lundh & Gøtzsche, 2008). The pre‐ and posttreatment data extracted from each study consisted of study design, mean age, number of patients of each treatment group, TMS parameters (number of sessions, target and localization method, frequency, intensity, total pulses, type of coil), dropouts and reasons, scale scores mean and standard deviation (*SD*), response and remission rates, and period of follow‐up. We contacted authors for additional data whenever necessary and we greatly appreciate the contributions of Dr. Zangen, Osuch, and Watts (Isserles et al., 2013; Osuch et al., 2009; Watts et al., 2012).

### 2.4 Quantitative analysis

The analysis was performed with Stata 15. The primary outcome was the improvement of each disorder measured by a validated scale. The effect sizes of controlled studies were determined with the mean differences of sham versus active TMS using pretreatment and posttreatment score changes. In studies with one group, the effect sizes were estimated with standardized mean difference of pre‐ and postscores, in which the subject is its own control. The denotation of effect size is the same independent of the study design and can be analyzed together (Borenstein, Hedges, Higgins, & Rothstein, 2009). All effect sizes were weighted with Hedges’ g, with a 95% confidence interval (CI) in a random effects model—which assumes variability across studies in terms of the effect size. In studies with three treatment groups, the active group with less effect was excluded. Heterogeneity between studies was assessed with the I‐square test (*I* ^2^). In case of moderate or high heterogeneity (*I* ^2^> 50%), a sensitivity analysis was done to determine the impact of each study on the results and a meta‐regression was performed to evaluate the influence of each TMS parameter at a time. For studies without the *SD* of the total score of the primary outcome, the largest similar *SD* found in other studies was repeated, according to the Cochrane Handbook for Systematic Review (Higgins & Green, 2011). Publication bias was evaluated by funnel plots of effect size versus standard error and by Egger's test (Egger, Davey Smith, Schneider, & Minder, 1997).

The studies were analyzed in four groups: SP, GAD, PD, and PTSD since there were no articles about TMS in SAD. Furthermore, the meta‐analysis was carried out only for GAD and PTSD since the other reviewed disorders do not have the minimum amount of studies and sample size needed to perform a meta‐analysis.

## 3 RESULTS

**Figure 1:**
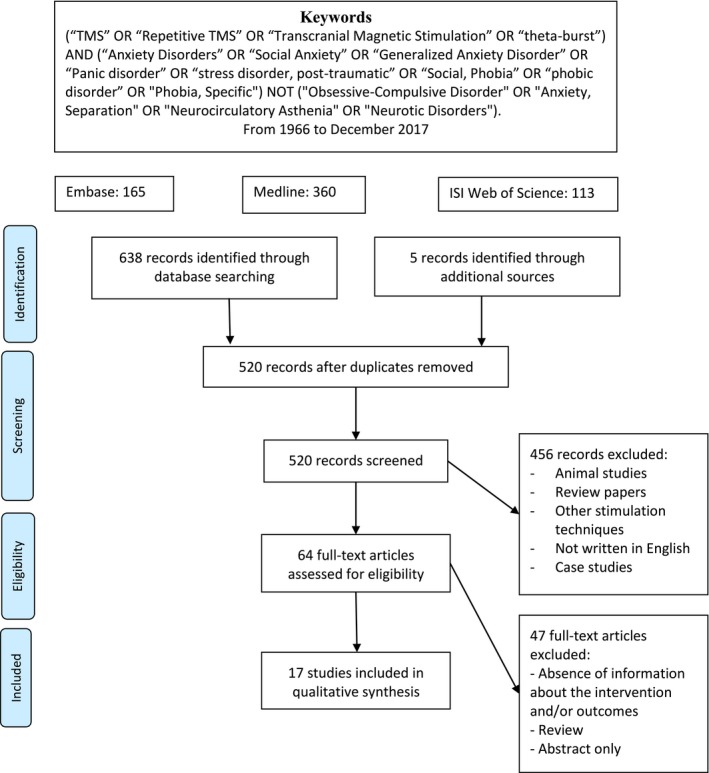
Flow chart of the search results and studies selection for the review of TMS and traumatic and anxiety disorders. From Moher et al. (2009)

A total of 643 references were found (165 in Embase, 360 in Medline, 113 in ISI Web of Science, and five through additional sources). Of those, 123 were duplicate references, and 37 were not in the English language. The remaining 483 references underwent a title and abstract analysis after which 419 were excluded. Finally, 64 articles were recovered for full‐text reading. After this process, only 17 articles met the inclusion criteria of articles that assessed TMS as a treatment for anxiety disorders or PTSD (nine PTSD, four GAD, two SP, and two PD) (Table 1). The meta‐analysis of SP and PD was not performed because of the small number of studies and sample size. Figure 1 depicts a flow chart of the search results and selection of studies.

**Table 1:** Number of included studies per psychiatric disorder and study design

Disorder	Double‐blind, randomized, sham‐controlled (*n*)	Single‐blind, randomized, sham‐controlled (*n*)	Open‐label	Retrospective
PTSD	6	0	1	2
GAD	2	0	2	0
SP	1	1	0	0
PD	2	0	0	0

### 3.1 TMS and generalized anxiety disorder

**Figure 2:**
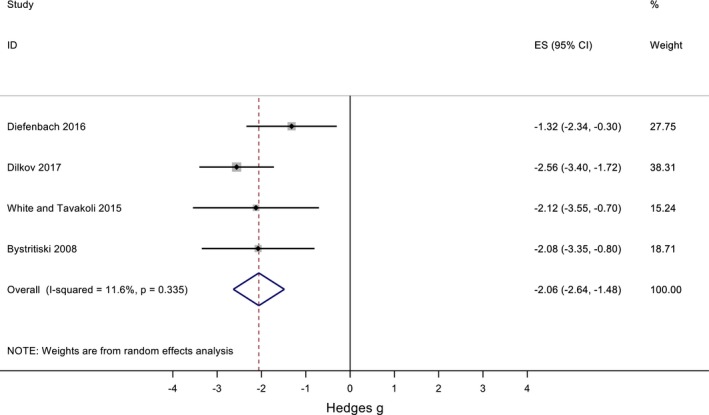
Forest plot of the 4 studies that evaluated rTMS as a treatment for GAD (2 RCT and 2 uncontrolled open‐label studies)

We identified a total of four studies that used TMS to treat GAD, of which two are randomized, double‐blind and sham‐controlled (Diefenbach et al., 2016; Dilkov, Hawken, Kaludiev, & Milev, 2017), and two are uncontrolled open‐trials (Bystritsky et al., 2008; White & Tavakoli, 2015). The rTMS parameters, questionnaires used, and method for target identification are in Table 2. Two studies applied low‐frequency (1 Hz) rTMS over the right dorsolateral prefrontal cortex (rDLPFC) (Bystritsky et al., 2008; Diefenbach et al., 2016). One study evaluated bilateral rTMS treatment in patients with comorbid GAD and MDD employing 1 Hz over the rDLPFC followed by 10 Hz over the left dorsolateral prefrontal cortex (lDLPFC) (White & Tavakoli, 2015). White and Tavakoli did not report the intensity applied on either side, nor the pulses delivered over the lDLPFC (White & Tavakoli, 2015). Last, one RCT applied 20 Hz, with 110% RMT over the rDLPFC (Dilkov et al., 2017). Figure 2 shows the weighted effect sizes of the studies.

**Table 2:** Therapeutic use of TMS in Generalized Anxiety Disorder

Study	Double‐blind (Y/N)	Randomized (Y/N)	Age (mean, years)	Active sample (*n*)	Sham sample (*n*)	Number of sessions	Target	Frequency	MT (%)	Total pulses	Drop outs (*n* due to AE)		Results		Follow‐up	Target identification method	Coil
											Active group	Sham	Response	Remission			
Diefenbach et al. (2016)	Y	Y	≥18 (44)	13 (9 completers)	12 (10 completers)	30	Right DLPFC	1 Hz	90% RMT	27,000	4	2	Active: 7/9 (77.8%) Sham: 2/10 (20%) *p* = 0.012	Active: 3/9 (33.3%) Sham: 1/10 (10%) *p* = 0.213	3 months Remission Active: 6/9 (66.7%) Sham: 0/10 Response Active: 7/9 (10%)	MNI coordinates	Sham: Neuronetics XPLOR Active: not reported
Dilkov et al. (2017)	Y	Y	23–57 (active: 34 ± 7/ sham: 38 ± 10)	25 (15 completers)	25 (25 completers)	20 (acute) + 5 (taper down)	Right DLPFC	20 Hz	110% RMT	90,000	10 (0)	0	Active group: 15/15 (100%) Sham: 3/25 (12%)	Active: 12/15 (80%)	1‐month follow‐up: active‐group remission = 15/15 (100%)	5 cm rule	Active: Figure of 8 Sham: 90^o^ from skull
White and Tavakoli (2015)	N	N	Mean: 42.46	13 GAD + MDD		24–36	Right + left DLPFC	1 Hz + 10 Hz	Not reported	rDLPFC: 24,000–36,000 lDLPFC: not reported	0		All responders also remitted	GAD ‐ 11/13 (84.6%) MDD – 10/13 (76.9%)	No	not reported	Not reported
Bystritsky et al. (2008, 2009)	N	N	18–56 (45.30 ± 12.1)	10		6 (twice/week, for 3 weeks)	Right DLPFC	1 Hz	90% RMT	5,400	0		8/10 (80%) Mean scores significantly decreased HAM‐A (*p* = 0.001) HAM‐D (*p* = 0.001) FDADS anxiety subscale (*p* = 0.000)	6/10 (60%)	6‐months (Bystritsky et al., 2008): sustained improvement. No significant difference to the end of rTMS treatment.	Activation across all task periods x resting state	Figure of 8

Note

DLPFC = dorsolateral prefrontal cortex; FDADS = Four‐dimensional anxiety and depression scale; GAD = generalized anxiety disorder; HAM‐A = Hamilton anxiety rating scale; HAM‐D = Hamilton depression rating scale; MDD = major depressive disorder; MT = motor threshold; *N* = no; RMT = resting motor threshold; rTMS = repetitive transcranial magnetic stimulation; Y = yes.

**Figure 3:**
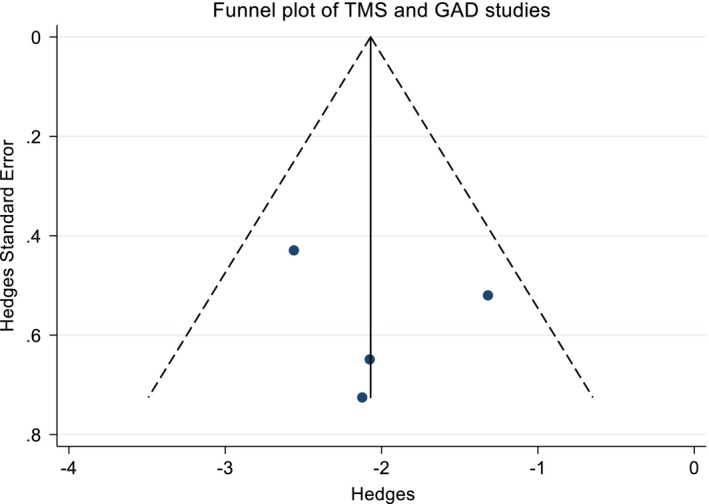
Funnel plot of the four studies that evaluated rTMS as a treatment for GAD

The overall effect size was −2.06 (95%CI: −2.64, −1.48), widely favoring active rTMS treatment. There was low heterogeneity (*I* ^2^ = 11.6%, *p* = 0.335); therefore, the difference between studies is by chance. Possible causes of publication bias were tested with the funnel plot (Figure 3), which showed no asymmetry (*p* = 0.705, Egger's test). Table 2 shows the reported dropouts and the number of dropouts due to side effects.

Three studies that evaluated the acute effects of rTMS in GAD, two RCT, and one uncontrolled open‐trial, followed the patients after 1, 3, or 6 months (Bystritsky, Kerwin, & Feusner, 2009; Diefenbach et al., 2016; Dilkov et al., 2017). Diefenbach et al. (2016) showed better results after a 3 month follow‐up than at the end of rTMS treatment; six of nine patients achieved remission compared to three at the end of rTMS. The number of responders remained the same. Dilkov et al. (2017), also found an increase in the remission rate of the active group, that reached 100% after 1‐month follow‐up. Bystritsky et al. (2009) reported the maintenance of the improvement after a 6‐month follow‐up without deterioration of questionnaire scores when compared to the end of his uncontrolled open‐label study (Bystritsky et al., 2008, 2009). As a group, these studies show that rTMS is a promising treatment for GAD.

### 3.2 TMS and posttraumatic stress disorder

**Figure 4:**
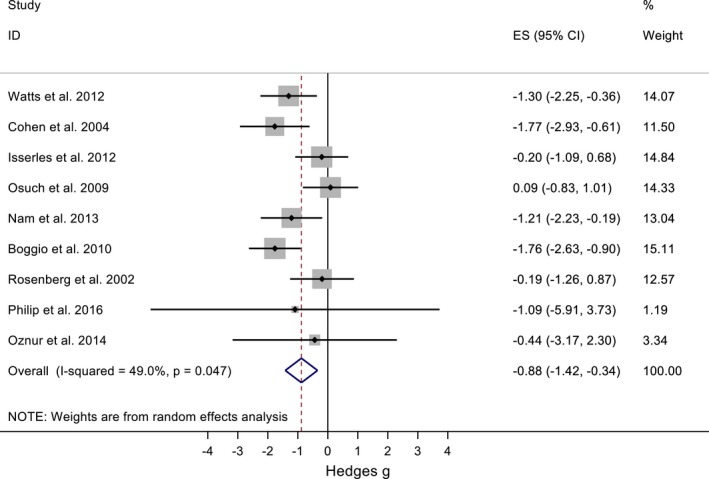
Forest plot of all nine PTSD and TMS studies

The treatment of PTSD with TMS is the most studied among the conditions of interest. Nine studies were included in this meta‐analysis (Boggio et al., 2010; Cohen et al., 2004; Isserles et al., 2013; Nam, Pae, & Chae, 2013; Osuch et al., 2009; Oznur et al., 2014; Philip, Ridout, Albright, Sanchez, & Carpenter, 2016; Rosenberg et al., 2002; Watts et al., 2012). Six trials are double‐blind, randomized, sham‐controlled, and one of these is a crossover. The other three are open‐label studies. The details of the study, including protocol parameters and validated questionnaires used are shown in Tables 3, 4, 5, 6. Figure 4 shows the unbiased weighted estimates of Hedges effect sizes with a random effects model. The overall effect size was −0.88 (95%IC: −1.42, −0.34), which favors TMS and suggests a medium treatment effect. The heterogeneity was low (I^2^=49.0%, *p* = 0.047). The funnel plot is symmetric (*p* = 0.992, Egger's test), suggesting that publication bias is unlikely. The reported dropouts and the amount of these that are due to side effects are in Tables 3, 4, 5, 6.

**Table 3:** Double‐blind, randomized, sham‐controlled studies of TMS in PTSD

Study	Age	Sham sample (*n*)	Group 2 Sample (*n*)	Group 3 Sample (*n*)	Number of sessions	Target	Frequency	MT	Total pulses	Drop outs (*n* due to AE)			Results Response	Scales	Follow‐up	Target identification method	Coil
										Sham	Act1	Act2					
Watts et al. (2012)	Sham: 57.8 (*SD* 11.8) Active: 54.0 (*SD* 12.3)	10	10		10	rDLPFC	1 Hz	90% VMT	4,000	0	0		rDLPFC (25% PCL – all clusters)> sham	CAPS PCL BDI STAI BNCE	2 months	4 cm anterior parasagittal and 2 cm lateral to the motor spot	Figure of 8
Nam et al. (2013)	Sham: 32.8 ± 6.9 years Active: 36.3 ± 8.8 years	9	7		15	rDLPFC	1 Hz	100% VMT	18,000	2 (0)	2		rDLPFC > sham (only for re‐experiencing)	CAPS	No	5 cm rule	Figure of 8
Boggio et al. (2010)	44.5 ± 4.4 years	10 (5 rDLPFC + 5 lDLPFC)	10 (rDLPFC)	10 (lDLPFC)	10	rDLPFC and lDLPFC	20 Hz	80% MT^a^	16,000	2	1	1	rDLPFC (36.9% PCL)> lDLPFC (23.1% PCL)> sham only for avoidance	PCL TOP−8 HAMD−28 HAMA	3 months	Not reported	Figure of 8
Cohen et al. (2004)	Sham = 42.8 14.8 (25–68), 1 Hz: 40.8 9.9 (24–52), 10 Hz: 41.8 11.4 (22–64)	6	8 (1 Hz)	10 (10 Hz)	10	rDLPFC	1 Hz and 10 Hz	80% VMT	1 Hz: 1,000 10 Hz: 4,000	2 (2)	1 Hz: 2(2) 10 Hz: 1 (0)		10 Hz (29.3% PCL and 39.0% TOP−8; 44.1% HAMA)> 1 Hz or sham (all PTSD clusters)	CAPS PCL TOP−8 HAMA, HAMD	14 days	5 cm rule	Circular
Isserles et al. (2013)	Sham: 40.4 _ 10.5, Group A: 49 _ 12.5, Group B: 40.5 _ 9.8,	9 PTSD + MDD (Group C: brief exp + sham)	9 (group A: active + traumatic virtual exposure)	8 (Group B: active + non‐traumatic virtual exposure)	12	mPFC	20 Hz	120% hand RMT	20,160	1 (1)	1 (0)	2 (2)	Sham: 0% Group A: 44% of patients (only re‐experiencing) Group B: 12.5% of patients	CAPS PSS‐SR HAMD−24 BDI‐II	No	Midline of the prefrontal cortex	H‐coil

Note

AE = adverse events; BDI – Beck Depression Inventory; BNCE – Brief neuropsychological cognitive examination; dTMS – deep TMS; CAPS ‐ Clinician Administered PTSD Scale; HAMA ‐ Hamilton Anxiety Rating Scale; HAMD ‐ Hamilton Depression Rating Scale; lDLPFC = left dorsolateral prefrontal cortex; mPFC = medial prefrontal cortex; MT = motor threshold; PCL = PTSD Checklist score; PSS‐SR = PTSD Symptom Scale—Self Report; PTSD = Posttraumatic Stress Disorder; rDLPFC = right dorsolateral prefrontal cortex; SADS ‐ Affective Disorders and Schizophrenia; STAI = State‐trait anxiety inventory; TOP‐8 – treatment outcome PTSD scale; VMT = visual motor threshold.

a Manuscript does not specify if it is resting or visual MT.

**Table 4:** Double‐blind, crossover, sham‐controlled studies of TMS in PTSD

Study	Age	Sample (*N*)	Cross‐over phase	Number of sessions	Target	Frequency	MT (%)	Total pulses	Drop outs 1st phase	Drop outs Cross over	Results Response	Scales	Rule	Scale	Coil
Osuch et al. (2009)	41.4 ± 12.3, (range 24–56)	9 PTSD + MDD	9 (same patients)	20	rDLPFC	1 Hz + exposure therapy	100% RMT	36,000	0	0	rDLPFC > sham (only for hyperarousal)	CAPS IES SADS HAMD	5 cm rule	CAPS	Figure of 8

Note

CAPS = Clinician Administered PTSD Scale; HAMD = Hamilton Depression Rating Scale; IES = Impact of Events Scale; MDD = major depressive disorder; MT = motor threshold; PTSD = Posttraumatic Stress Disorder; rDLPFC = right dorsolateral prefrontal cortex; SADS = Affective Disorders and Schizophrenia; VMT = visual motor threshold.

**Table 5:** Open‐label studies of TMS in PTSD

Study	Randomized	Age	Group 1 Sample (*n*)	Group 2 Sample (*n*)	Number of sessions	Target	Frequency	MT	Total pulses	Drop outs	Results Response	Scale	Follow‐up	Rule	Coil
Rosenberg et al. (2002)	Y	54.8 ± 9.1	6 PTSD + MDD	6 PTSD + MDD	10	lDLPFC	1 or 5 Hz	90% RMT	6,000	3 of 15 (1 due to AE)	MISS score: reduction of 4% Depression 1 Hz‐ 4/6 patients (67%) 5 Hz – 5/6 patients (83%)	MISS	2 months: MISS score: reduction of 6% (*p* = 0.02) Depression 1 Hz‐ 3/6 (50%) 5 Hz – 3/6 (50%)	4 cm anterior parasagittal and 2 cm lateral to the motor spot	Figure of 8

Note

AE = adverse events; lDLPFC = left dorsolateral prefrontal cortex; MDD = major depressive disorder; MISS = Mississippi Scale of Combat Severity; MT = motor threshold; PTSD = Posttraumatic Stress Disorder; RMT = resting motor threshold; Y = yes.

**Table 6:** Retrospective studies of TMS in PTSD

Study	Age	Sample	Number of sessions	Target	Frequency	MT	Total pulses	Drop outs	Results	Scale	Rule	Coil
Philip et al. (2016)	58.1 ± 13.9 years	10 PTSD + MDD	36	lDLPFC	5 Hz	120%^a^	108,000 to 129,000	4 (0)	40% (4/10) for PTSD, 50% (5/10) for MDD	PCL QIDS	F3 EEG 10/20	Not reported
Oznur et al. (2014)	28.7 (±3.3) (20–40)	20 Male combat‐related PTSD	20	rDLPFC	1 Hz	80% RMT	12,000	NA	Improvement only of hyperarousal symptoms	IES BDI BAI	5 cm rule	Figure of 8

Note

BAI – Beck Anxiety Inventory; BDI – Beck Depression Inventory; F3 EEG 10/20 = F3 position of 10–20 system of electroencephalogram electrode placement; IES ‐ Impact of Events Scale; lDLPFC = left dorsolateral prefrontal cortex; MDD = major depressive disorder; MT = motor threshold; PCL – PTSD checklist; PTSD = Posttraumatic Stress Disorder; QIDS – Quick inventory of depressive symptomatology; rDLPFC = right dorsolateral prefrontal cortex; RMT = resting motor threshold.

a Manuscript does not specify if it is resting or visual MT.

All studies applied 1–20 Hz rTMS with traditional figure‐of‐eight coils to either the right or left DLPFC or both, with the exception of one study that evaluated the effect of dTMS to the medial PFC (mPFC) (Isserles et al., 2013). Six studies administered 10–15 sessions (Boggio et al., 2010; Cohen et al., 2004; Isserles et al., 2013; Nam et al., 2013; Rosenberg et al., 2002; Watts et al., 2012), two administered 20 sessions (Osuch et al., 2009; Oznur et al., 2014), and one 36 sessions (Philip et al., 2016). Concerning the sample characteristics, two studies assessed combat‐related PTSD, and in one of these studies, all patients had a history of substance abuse (Oznur et al., 2014; Rosenberg et al., 2002). Also, four studies evaluated comorbid PTSD and MDD (Isserles et al., 2013; Osuch et al., 2009; Philip et al., 2016; Rosenberg et al., 2002). Three of the RCT consisted of three treatment groups (Boggio et al., 2010; Cohen et al., 2004; Isserles et al., 2013). One study compared 20 Hz rTMS over the right or left DLPFC against sham, and another study compared 1–10 Hz over the rDLPFC (Boggio et al., 2010; Cohen et al., 2004). High frequency over the rDLPFC showed better results in both studies. Moreover, the study of Isserles et al. (2013) compared active and sham 20 Hz dTMS to the mPFC combined with exposure to images of traumatic and nontraumatic events. Response was defined as an improvement of at least 50% in CAPS score. The response rate was 44% in the active‐dTMS/traumatic images‐group while in the active‐dTMS/nontraumatic images‐group was 12.5% and, in the sham‐dTMS/traumatic images‐group was 0% (Isserles et al., 2013). PTSD is characterized by intrusion or re‐experiencing, avoidance, and hyperarousal clusters of symptoms (Ruggiero, Del Ben, Scotti, & Rabalais, 2003). In this study, they observed improvement of re‐experiencing symptoms in the active‐dTMS/traumatic images‐group (Isserles et al., 2013).

Three studies reported an improvement of all clusters of symptoms (Cohen et al., 2004; Philip et al., 2016; Watts et al., 2012), two studies reported an improvement only on the hyperarousal cluster (Osuch et al., 2009; Oznur et al., 2014), two studies reported an improvement only on the re‐experiencing cluster (Isserles et al., 2013; Nam et al., 2013), and one study reported an improvement only on avoidance (Boggio et al., 2010). The two studies that applied rTMS over the lDLPFC in PTSD/MDD patients showed improvement of depressive symptoms as well (Philip et al., 2016; Rosenberg et al., 2002).

Four studies evaluated patients at follow‐up intervals of 14 days (Cohen et al., 2004), 2 months (Rosenberg et al., 2002; Watts et al., 2012), or 3 months (Boggio et al., 2010). Three of these studies showed that there was a loss of improvement in PTSD symptoms at follow‐up relative to the end of treatment despite the improvement from baseline (Boggio et al., 2010; Cohen et al., 2004; Watts et al., 2012). The one other study, which found that patients had improvements in MDD symptoms but not PTSD symptoms posttreatment, also found decreased depressive symptom improvement 2 months after the end of rTMS treatment (Rosenberg et al., 2002).

**Figure 5:**
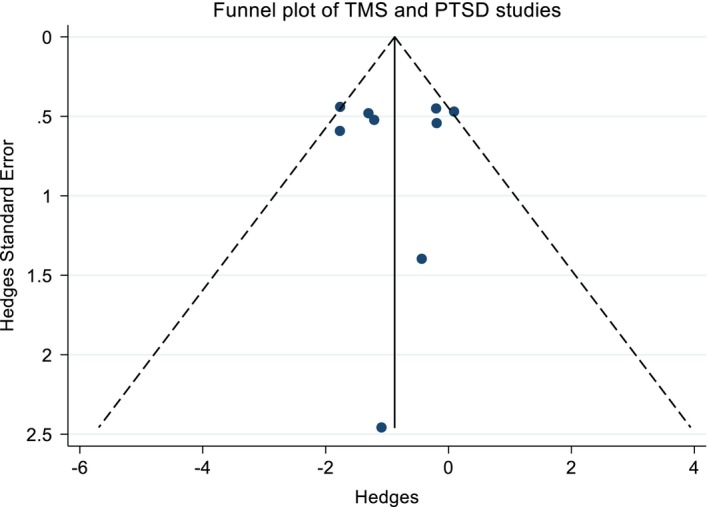
Forest plot for the meta‐analysis of the treatment of PTSD with TMS

Tables 3, 4, 5 and 6 summarize studies that have evaluated the application of TMS in PTSD. Figure 5 depicts a forest plot for the meta‐analysis evaluating TMS as a treatment for PTSD.

**Table 7:** Therapeutic use of TMS in panic disorder

Study	Double‐blind (Y/N)	Randomized (Y/N)	Age (mean, years)	Active sample (*n*)	Sham sample (*n*)	Control sample (*n*)	Number of sessions	Target	Frequency	MT (%)	Total pulses	Drop outs (*n* due to adverse events)	Results	Follow‐up	Task	Target identification method	Coil
Deppermann et al. (2014)	Y	Y	Active: 19–63 (37.6) Sham: 22–56 (36.3) HC: 19–64 (33.4)	22 PD	22 PD	23 HC	PD: 15 iTBS + 3 weekly psychoeducation sessions HC: only 3 weekly psychoeducation sessions	Left DLPFC	Standard iTBS	80% RMT	9,000 (only PD patients)	not reported	No significant changes in PAS, HAMA and CAQ between groups		No	F3 EEG 10–20	Figure of 8
Mantovani et al. (2013)	Y	Y	Active: 40.27 ± 10 Sham: 39.87 ± 13.3	Phase 1: 12 (11 completers) PD + MDD Phase 2:9	Phase 1:13 (10 completers) PD + MDD Phase 2:8		20	Right DLPFC	1 Hz	110% RMT	36,000	Phase 1: Active: 1 (0) Sham: 3 (0)	Phase 1: PD response rate: 6/12 (50%) (active) x 1/13 (7.7%) (sham). MDD: 3/12 (25%) Phase 1 + 2 (open‐label): PD response rate: 8/12 (67%) MDD response rate: 6/12 (50%)	Phase 1: PD remission rate: 3/12 (25%) MDD: 1/12 (8.3%) Phase 1 + 2 (open‐ label): PD remission rate: 6/12 (50%) MDD remission rate: 2/12 (16.7%)	6 months: sustained improvement (7/9 in remission)	5 cm rule	Figure of 8

Note

CAQ: cardiac anxiety questionnaire; DLPFC = dorsolateral prefrontal cortex; EEG = electroencephalogram; fNIRS = functional near‐infrared spectroscopy; HAM‐A: Hamilton Anxiety Rating Scale; HC = health controls; iTBS = intermittent theta‐burst stimulation; MT = motor threshold; PAS = Panic and Agoraphobia Scale, PD = panic disorder; Y = yes.

**Table 8:** Therapeutic use of TMS in specific phobia

Study	Double‐blind (Y/N)	Randomized (Y/N)	Mean age/*SD* (years)	Active sample (*n*)	Sham sample (*n*)	Number of sessions	Target	Frequency	MT (%)	Total pulses	Drop outs		Results	Task	Follow‐up	Target identification method	Coil
											Active	Sham					
Herrmann et al. (2017)	Y	Y	Active group 43.2 ± 12.6 Sham group 46.6 ± 13.7	Acrophobia: 20	Acrophobia: 19	2 sessions with an inter session interval of 1 week	vmPFC	10 Hz	100% RMT	3,120	1 (reasons not reported)	2 (reasons not reported)	Active > sham for anxiety (*p* < 0.05) and avoidance ratings (*p* < 0.05)	Height scenario virtual reality	3 months	MNI coordinates based on mPFC activation	Round MMC−140 Parabolic or MC‐p‐B70 placebo
Notzon et al. (2015)	Single‐blind	Y	26.46 ± 8.47	Spider phobia: 21 HC: 19	Spider phobia: 20 HC: 23	1	left DLPFC	Standard iTBS	80% RMT	600 (iTBS)	not reported	not reported	No iTBS effect in acute anxiety or disgust	Virtual reality provoked anxiety	No	F3 EEG 10–20	Not reported

Note

DLPFC = dorsolateral prefrontal cortex; EEG = electroencephalogram; FSQ = fear of spiders questionnaire; HC = health controls; iTBS = intermittent theta burst stimulation; mPFC = medial prefrontal cortex; MT = motor threshold; RMT = resting motor threshold; *SD*: standard deviation; SPQ = Spider phobia questionnaire; vmPFC = ventromedial prefrontal cortex; Y = yes.

### 3.3 TMS and panic disorder

Two double‐blind, randomized, sham‐controlled trials evaluated the efficacy of rTMS or iTBS, respectively, as a treatment of PD (Deppermann et al., 2014; Mantovani, Aly, Dagan, Allart, & Lisanby, 2013). One study evaluated the treatment of comorbid PD and MDD with rTMS (Mantovani et al., 2013). This study enrolled 25 patients, randomized to active (*n* = 12) or sham (*n* = 13) rTMS. They applied 1 Hz, at 110% RMT, and 1,800 pulses/session, over the rDLPFC, for 4 weeks. After the last week of treatment, patients in active rTMS had a significant improvement in their PD but not in their MDD. This study was followed by four additional weeks of an open‐label treatment in which patients in the sham group could undergo active treatment and patients in the active group could receive additional treatment. After this second phase, patients continued to improve from PD and improved from MDD. Subsequently, at a 6‐month follow‐up, patients showed sustained improvement of both disorders (Mantovani et al., 2013).

The other study evaluated whether iTBS associated with psychoeducation could ameliorate clinical symptoms, verbal fluency, and brain activity of PD patients (Deppermann et al., 2014). This study assessed 44 patients with PD and 23 healthy controls. PD patients were equally randomized to sham or standard iTBS. Both PD groups underwent 15 weekday iTBS sessions. All participants completed a verbal fluency task during functional near‐infrared spectroscopy (NIRS) and three‐weekly group psychoeducation sessions. The healthy controls had not undergone rTMS. In the end, both active and sham rTMS groups showed substantial improvement of PD symptoms, without significant difference between groups. There were no improvements in prefrontal hypoactivity or verbal fluency following iTBS (Deppermann et al., 2014).

### 3.4 TMS and specific phobia

We did not find any studies for SP with more than two treatment sessions. However, due to the peculiar features of the disorder with acute exacerbations that can be predicted in some situations, patients could benefit from short‐lasting effects of stimulation. Two studies used single‐session paradigms with a translational (not therapeutic) aim that are informative in the context of this review. These studies evaluated rTMS or excitatory intermittent theta burst stimulation (iTBS) as a treatment for SP (Herrmann & Ebmeier, 2006; Notzon et al., 2015). Notzon et al. (2015) evaluated the effects of one iTBS session on virtual reality‐provoked anxiety in 41 patients with spider phobia and 42 healthy controls randomized to active or sham iTBS; however, they measured the fear of spiders (SPQ), anxiety (ASI), and disgust sensitivity (DS) using questionnaires. They stimulated the lDLPFC using 600 pulses and the traditional iTBS protocol (50 Hz triplets every 200 ms for 2 s on and an intertrain interval of 8 s) with a pulse intensity of 80% of the resting motor threshold (RMT). One session of iTBS showed no improvement.

Previous studies showed the importance of the ventromedial prefrontal cortex (vmPFC) in fear extinction (Herrmann & Ebmeier, 2006). Since this brain area is too deep to be directly modulated by TMS, a research group used the strategy to indirectly stimulate this region through FPz, according to the electroencephalography (EEG) 10–20 system. This position had been identified as the center of the mPFC activation cluster by an increase of oxygenated hemoglobin during extinction of conditioned fear measured by NIRS in a prior study (Guhn et al., 2012). Herrmann and Ebmeier (2006) studied the effect of active (*n* = 20) or sham (*n* = 19) rTMS applied before a virtual reality exposure to heights in two groups of individuals diagnosed with acrophobia. The protocol consisted of two active sessions of 20 min of rTMS with 10 Hz, at 100% RMT, 4 s on and 26 s off, with 1560 pulses per session, and the sessions were 1 week apart. At the end, anxiety (*t* = 37, 2.33, *p* < 0.05) and avoidance ratings (*t* = 37, 2.34, *p* < 0.05) decreased in the active group (Herrmann & Ebmeier, 2006).

### 3.5 Side effects of TMS

Ten of the 17 studies (59%) included in this meta‐analysis presented adverse events (Boggio et al., 2011; Cohen et al., 2004; Diefenbach et al., 2016; Dilkov et al., 2017; Herrmann & Ebmeier, 2006; Isserles et al., 2013; Mantovani et al., 2013; Nam et al., 2013; Notzon et al., 2015; Rosenberg et al., 2002). Most of the side effects were mild to moderate. However, two studies reported a single generalized tonic‐clonic seizure (Dilkov et al., 2017; Isserles et al., 2013). Both of these studies applied 20 Hz. One study used rTMS with 20 trains of 9 s, 51 s intertrain intervals, 110% RMT, 3,600 pulses/session, with a figure‐of‐eight coil over the rDLPFC (Dilkov et al., 2017). A train of 9 s is long and may have contributed to the seizure. The other study used dTMS with 42 trains of 2 s, 20 s intertrain intervals, 120% RMT, 1680 pulses/session, with a H‐coil over the mPFC (Isserles et al., 2013). This protocol parameters are in the upper limit of the parameters currently used for dTMS. Neither described clinical characteristics that could explain a higher risk of seizure.

Adverse events in patients who underwent active TMS were headache, neck pain, scalp pain, tingling, sleepiness, facial twitch, and impaired cognition during treatment. A PTSD study reported two patients with manic episodes: one patient in the 1 Hz‐group and another in the 10 Hz‐group (Cohen et al., 2004). Few studies reported the adverse events of the sham group separately, but these included neck and scalp pain, headache, impaired cognition, dizziness, sleepiness, and discomfort with treatment and the study schedule (Boggio et al., 2010; Diefenbach et al., 2016; Isserles et al., 2013; Mantovani et al., 2013; Nam et al., 2013). One PD study reported hearing impairment, mainly in the sham group (Mantovani et al., 2013). Adverse events are described in Table 9.

**Table 9:** Occurrence of adverse events in TMS treatment of anxiety disorders and PTSD

Adverse Event *n* (treatment‐group)	Author (year)									
	GAD		PTSD					PD	SP	
	Diefenbach (2016)^a^	Dilkov (2017)	Nam (2013)	Boggio (2010)^a^, ^b^	Cohen (2004)	Isserles (2013)	Rosenberg (2002)^b^	Mantovani (2013)	Notzon (2015)	Herrmann (2017)
Scalp pain	X					1 (active‐dTMS/traumatic images)		X^a^	X (standard ‐iTBS)	1 (sham)
Facial twitch		X								
Headache			3 (1 Hz‐group)	X	13^b^	X	1	X^a^		7 (10 Hz‐group)
			2 (sham)							
Discomfort						1 (active ‐dTMS/traumatic images)			X (standard ‐iTBS)	
Seizure		1 (20 Hz‐group)				1 (active ‐dTMS/traumatic images)				
Dizziness		3 (20 Hz‐group)	1 (1 Hz‐group)	X	1 (10 Hz‐group)					1 (10 Hz‐group)
			1 (sham)							
Cognitive impairment			1 (sham)					X^a^		
Neck pain				X	2 (10 Hz‐group)			X^a^		2 (sham)
Sleepiness				X						
Manic episode					1 (10 Hz‐group)					
					1 (1 Hz‐group)					
Rage attack					1^b^					
Ear discomfort or hearing impairment					2 (<1 min duration)^b^			X (1 Hz‐group < sham)		
Increased anxiety						1 (active ‐dTMS/traumatic images)				
Uncomfortable with treatment and study schedule						1 (active ‐dTMS/traumatic images)				

X = adverse event occurred but the study did not report on what number of patients; dTMS = deep transcranial magnetic stimulation; GAD = generalized anxiety disorder; iTBS = intermittent Theta burst stimulation; PD = panic disorder; PTSD = posttraumatic stress disorder; SP = specific phobia.

a Article reported similar frequency of adverse events between groups.

b Article reported the adverse events of both active groups together.

Another critical issue is to evaluate the percentage of patients who dropped out due to adverse events. A quarter of the studies reported the reasons for dropouts: the minority of dropouts was due to adverse events and no studies reported treatment ineffectiveness as a reason for dropouts. The causes of dropouts varied from withdrawal or improvement of the disorder before starting treatment, to impossibility to determine the motor threshold, and technical error (Cohen et al., 2004; Dilkov et al., 2017; Rosenberg et al., 2002). Considering studies that evaluated TMS as a treatment for PTSD, one study reported two dropouts: one because of increased anxiety and one due to unease (Isserles et al., 2013), and another reported one dropout in a PTSD sample due to marked headache (Rosenberg et al., 2002). Therefore, there was no difference in the dropout rate due to adverse events between active and sham TMS treatments. However, only 24% of the studies reported in detail the reasons for dropouts per treatment group.

## 4 DISCUSSION

This review analyzes existing studies that evaluated TMS as a treatment for anxiety disorders or PTSD. Regarding GAD, the overall effect size largely favors TMS treatment (Bystritsky et al., 2008; Diefenbach et al., 2016; Dilkov et al., 2017; White & Tavakoli, 2015). Three of the four studies targeted the rDLPFC, two with 1 Hz inhibitory TMS and one with 20 Hz excitatory TMS (Bystritsky et al., 2009; Diefenbach et al., 2016; Dilkov et al., 2017). The other study associated 1Hz‐rTMS over the rDLPFC and 10Hz‐rTMS over the lDLPFC since the sample had comorbid GAD and MDD, and achieved high remission rates in both disorders (GAD: 84.6%, MDD: 76.9%) (White & Tavakoli, 2015). The only study that used 20 Hz on the right side (as opposed to the usual 1 Hz) and 110% RMT presented the best response and remission rates, and highest effect size (Dilkov et al., 2017). Three GAD studies reported follow‐ups from 1 to 6 months. The 6‐month follow‐up showed sustained improvement and the follow‐ups of 1 and 3 months showed that patients were better when compared to the end of TMS treatment (Bystritsky et al., 2009; Diefenbach et al., 2016; Dilkov et al., 2017).

In relation to PTSD, the overall effect size was also large (Boggio et al., 2010; Cohen et al., 2004; Isserles et al., 2013; Nam et al., 2013; Osuch et al., 2009; Oznur et al., 2014; Philip et al.., 2017; Rosenberg et al., 2002; Watts et al., 2012). Considering the four PTSD studies that have larger effect sizes and small variability (all of these randomized, sham‐controlled trials), there are indications that the rDLPFC is a better target to treat PTSD and anxiety symptoms when compared to the lDLPFC. Furthermore, two of these four studies applied high‐frequency rTMS (10 and 20 Hz) over the rDLPFC and compared with low frequency over the rDLPFC or high frequency over the lDLPFC and, in both studies, high‐frequency rTMS (10 and 20 Hz) over the rDLPFC showed greater improvement (Boggio et al., 2010; Cohen et al., 2004). The only trial that used dTMS could not demonstrate a substantial treatment effect of 12 sessions over the mPFC (Isserles et al., 2013). Therefore, further studies could assess the efficacy of dTMS with more sessions and over other cortical areas.

In three of the four studies that treated patients with comorbid MDD, which affects half of patients with PTSD, there was no significant improvement of depressive symptoms. The study that achieved response rates of 40% for PTSD and 50% for MDD applied 36 rTMS sessions while the other studies applied 10–20 sessions. The standard TMS course as a treatment for MDD consists of at least 30 sessions. Therefore, it is likely that a greater number of sessions could assign better results for both MDD and PTSD. The three PTSD studies that followed patients from 14 days to 3 months already found deterioration of PTSD improvement relative to the end of TMS treatment, despite remaining better when compared to baseline (Boggio et al., 2010; Cohen et al., 2004; Rosenberg et al., 2002; Watts et al., 2012).

Considering both GAD and PTSD outcomes, studies that targeted the rDLPFC with high frequency showed better results (Boggio et al., 2010; Dilkov et al., 2017; Isserles et al., 2013). In general, these results suggest that rDLPFC rTMS might have therapeutic activity in GAD and PTSD and that both high‐ and low‐frequencies work. Therefore, despite the low‐frequency rTMS being the standard treatment for rDLPFC indications, including MDD, anxious depression, and MDD with anxiety comorbidities, the use of high‐frequency TMS to the rDLPFC may have more empirical support. Nevertheless, this hypothesis needs further validation. Additionally, GAD follow‐ups showed that TMS effect may increase beyond the end of treatment while in PTSD patients the effect had already decreased 14 days after the last session. It is possible that a greater number of sessions in PTSD treatment would promote longer‐lasting improvement. Notably, these differences may be due to the pathophysiological differences of the two disorders, which would require unique approaches to induce therapeutic plasticity (Camprodon & Pascual‐Leone, 2016). Appropriately powered randomized controlled trials should be considered to empirically confirm and validate these meta‐analytical conclusions.

SP is still neglected, so almost no conclusions can be drawn except that treatments with more than one session should be used with intensities of at least 100% MT. Similarly, it is difficult to make assumptions on the use of TMS as a treatment for PD based on two small and heterogeneous trials. However, there are indications that 1 Hz over the rDLPFC may work with intensities higher than 100% RMT. On the other hand, future studies may clarify whether the failure of PD treatment on the left side was due to laterality or the iTBS technique.

TMS seems to be safe and well tolerated by patients with anxiety disorders or PTSD, although we found major gaps in the reports of these data. Two thirds of the studies in this meta‐analysis reported the side effects but four of these studies just reported the types of side effects without mention of frequency or relation to treatment group. This is an important gap that highlights the need to systematically assess and report adverse events with validated questionnaires. This practice would allow for a comparison across treatment conditions and risk‐benefit analysis.

## 5 LIMITATIONS

One limitation of our meta‐analysis is that 12 of the 17 studies were performed with small sample sizes of less than 20 subjects in each group. Moreover, across the reviewed studies, there is an absence of uniformity on the study design and how outcomes are measured and reported. These factors make it difficult to generalize the results, although meta‐analytical approaches exist and were used. Furthermore, there may have been language bias since only English studies were included. However, it is unlikely that this bias would not interfere with the results of the meta‐analysis. Finally, the lack of reporting of adverse events restricts the evaluation of safety and tolerability.

## 6 CONCLUSION

While there are still limited data on the effectiveness of TMS in anxiety or trauma‐related disorders (few studies, with small samples and diverse study designs and protocols), a number of trials have been published particularly for GAD and PTSD. Our meta‐analysis concludes an overall positive therapeutic effect of TMS for these two conditions. These results suggest (but do not prove) an advantage of right over lDLPFC stimulation, and the possible therapeutic advantage of high‐frequency stimulation to the rDLPFC. Based on the studies that reported side effects, TMS demonstrated to be safe and well tolerated in the treatment of anxiety disorders and PTSD but reports of side effects were inconsistent. In summary, the result of this meta‐analysis confirms the therapeutic potential and safety of TMS for GAD and PTSD and generates some hypotheses for upcoming prospective, larger, and appropriately powered randomized controlled trials to confirm these results.

## DISCLOSURE STATEMENT

AKG receives research support from NIMH**.** AAN reports the following disclosures: *Consultant ‐* Abbott Laboratories, Alkermes, American Psychiatric Association, Appliance Computing Inc. (Mindsite), Basliea, Brain Cells, Inc., Brandeis University, Bristol Myers Squibb, Clintara, Corcept, Dey Pharmaceuticals, Dainippon Sumitomo (now Sunovion), Eli Lilly and Company, EpiQ, L.P./Mylan Inc., Forest, Genaissance, Genentech, GlaxoSmithKline, Healthcare Global Village, Hoffman LaRoche, Infomedic, Intra‐Cellular Therapies, Lundbeck, Janssen Pharmaceutica, Jazz Pharmaceuticals, Medavante, Merck, Methylation Sciences, NeuroRx, Naurex, Novartis, PamLabs, Parexel, Pfizer, PGx Health, Otsuka, Ridge Diagnostics Shire, Schering‐Plough, Somerset, Sunovion, Takeda Pharmaceuticals, Targacept, and Teva**;** consulted through the MGH Clinical Trials Network and Institute (CTNI) for Astra Zeneca, Brain Cells, Inc, Dianippon Sumitomo/Sepracor, Johnson and Johnson, Labopharm, Merck, Methylation Science, Novartis, PGx Health, Shire, Schering‐Plough, Targacept and Takeda/Lundbeck Pharmaceuticals, NeuroRx Pharma, Pfizer, Physician's Postgraduate Press, Inc. *Grants/Research support ‐* American Foundation for Suicide Prevention, AHRQ, Brain and Behavior Research Foundation, Bristol‐Myers Squibb, Cederroth, Cephalon, Cyberonics, Elan, Eli Lilly & Company, Forest, GlaxoSmithKline, Intra‐Cellular Therapies, Janssen Pharmaceuticals, Lichtwer Pharma, Marriott Foundation, Mylan, NIMH, PamLabs, Patient Centered Outcomes Research Institute (PCORI), Pfizer Pharmaceuticals, Shire, Stanley Foundation, Takeda/Lundbeck, and Wyeth‐Ayerst. *Honoraria ‐* Belvoir Publishing, University of Texas Southwestern Dallas, Brandeis University, Bristol‐Myers Squibb, Hillside Hospital, American Drug Utilization Review, American Society for Clinical Psychopharmacology, Baystate Medical Center, Columbia University, CRICO, Dartmouth Medical School, Health New England, Harold Grinspoon Charitable Foundation, IMEDEX, International Society for Bipolar Disorder, Israel Society for Biological Psychiatry, Johns Hopkins University, MJ Consulting, New York State, Medscape, MBL Publishing, MGH Psychiatry Academy, National Association of Continuing Education, Physicians Postgraduate Press, SUNY Buffalo, University of Wisconsin, University of Pisa, University of Michigan, University of Miami, University of Wisconsin at Madison, APSARD, ISBD, SciMed, Slack Publishing and Wolters Klower Publishing, ASCP, NCDEU, Rush Medical College, Yale University School of Medicine, NNDC, Nova Southeastern University, NAMI, Institute of Medicine, CME Institute, ISCTM, World Congress on Brain Behavior and Emotion, Congress of the Hellenic Society for Basic and Clinical Pharmacology, ADAA.* Stock ‐* Appliance Computing, Inc. (MindSite); Brain Cells, Inc., Medavante**.** *Copyrights* ‐ Clinical Positive Affect Scale and the MGH Structured Clinical Interview for the Montgomery Asberg Depression Scale exclusively licensed to the MGH Clinical Trials Network and Institute (CTNI).* Speaker Bureaus* ‐ none since 2003. JAC is a scientific advisor for Apex Neuroscience. GK has received research support from Astra‐Zeneca, Bristol‐Myers Squibb Company, Cephalon, Elan Pharmaceuticals, Eli Lilly & Company, Forest Pharmaceuticals Inc., GlaxoSmithkline, Sanofi/Synthelabo, Sepracor Inc., Pfizer Inc, UCB Pharma, and Wyeth‐Ayerst Laboratories, Agency for Healthcare Research and Quality (AHRQ) Grant R01 HS019371‐01, and Takeda Pharmaceuticals. He has been an advisor or consultant for Astra‐Zeneca, Cephalon, Eli Lilly & Company, Forest Pharmaceuticals Inc., GlaxoSmithkline, Janssen Pharmaceutica, Pfizer Inc, Sepracor Inc., UCB Pharma, and Wyeth‐Ayerst Laboratories. GK has been a speaker for Astra‐Zeneca, Forest Pharmaceuticals Inc., GlaxoSmithkline, Sepracor Inc., and Wyeth‐Ayerst Laboratories. The other authors have no conflicts of interest to declare.
